# Consistent *in vitro* activity of sulbactam-durlobactam against genetically diverse carbapenem-resistant *Acinetobacter baumannii* in a high-endemic Brazilian setting

**DOI:** 10.1128/aac.00109-26

**Published:** 2026-06-12

**Authors:** Gabriela da Silva Collar, Luana Silva Dornelles, Júlia Cover Salvador, Jaqueline Becker Pinto, Dariane Castro Pereira, Patrícia Orlandi Barth, João Vitor Barboza Cardoso, William Latosinski Matos, Mariana Preussler Mott, Afonso Luís Barth, Andreza Francisco Martins, Juliana Caierão

**Affiliations:** 1 Laboratório de Pesquisa em Bacteriologia Clínica, Universidade Federal do Rio Grande do Sul 28124, Porto Alegre, Brazil; 2 Programa de Pós-graduação em Ciências Farmacêuticas, Universidade Federal do Rio Grande do Sul 28124, Porto Alegre, Brazil; 3 Laboratório Central do Hospital Nossa Senhora da Conceição, Grupo Hospitalar Conceição 125207, Porto Alegre, Brazil; 4 Laboratório de Pesquisa em Resistência Antimicrobiana, Hospital de Clínicas de Porto Alegre 37895, Porto Alegre, Brazil; 5 Serviço de Diagnóstico Laboratorial, Hospital de Clínicas de Porto Alegre 37895, Porto Alegre, Brazil; 6 Programa de Pós-graduação em Ciências Médicas, Universidade Federal do Rio Grande do Sul 28124, Porto Alegre, Brazil; 7 Núcleo de Bioinformática, Hospital de Clínicas de Porto Alegre 37895, Porto Alegre, Brazil; University of Pennsylvania Perelman School of Medicine, Philadelphia, Pennsylvania, USA

**Keywords:** Latin America, carbapenem-resistant, sulbactam-durlobactam, *Acinetobacter baumannii*

## Abstract

Carbapenem-resistant *Acinetobacter baumannii* (CRAb) causes high-mortality infections, with limited treatment options. We evaluated sulbactam-durlobactam activity against 94 isolates from Brazil. Durlobactam markedly reduced sulbactam MICs, restoring susceptibility in all CRAb. They belonged to seven STs, mainly ST730 and ST79. Despite genetic and phenotypic diversity, including distinct carbapenemase genes (particularly *bla*_OXA-23-like_) and the presence of PBP3 mutations, activity remained consistent. These findings support sulbactam-durlobactam as an effective option against CRAb in this high-endemic setting.

## INTRODUCTION

Carbapenem-resistant *Acinetobacter baumannii* (CRAb) frequently causes hard-to-treat infections with elevated mortality (1, 2), particularly in high-endemic settings such as Brazil (3, 4). Sulbactam-durlobactam has been recommended as a preferred therapeutic option against CRAb (5, 6) with promising global results (6–16).

However, data from Latin America remain limited, underscoring the urgent need for regional evaluation. This observational, cross-sectional study aimed to assess the *in vitro* activity of sulbactam-durlobactam against CRAb from Brazil (Research Ethics Committee approval number 84077624.9.00005530).

It included 94 CRAb from patients admitted to a tertiary healthcare complex (geographically distinct buildings) in Porto Alegre, Southern Brazil. They were prospectively/consecutively (*n* = 65; December 2024–February 2025) and retrospectively (*n* = 29; January 2023–April 2024) recovered. Species identification was assessed by MALDI-TOF MS (Bruker Daltonics).

Disk diffusion was executed following BrCAST/EUCAST (17, 18). Broth microdilution (BMD) (19) was performed in duplicate to determine the minimum inhibitory concentration (MIC) of meropenem (0.5–256 mg/L), polymyxin B (0.25–64 mg/L), and sulbactam (0.125–256 mg/L), alone or combined with a fixed concentration of durlobactam (4 mg/L). Results were interpreted following CLSI (20).

The presence of *bla*_OXA-23-like_ and *bla*_OXA-24/40-like_ was determined by multiplex PCR (21). Population diversity was phenotypically assessed for all isolates using Fourier transform-infrared (FT-IR) spectroscopy (IR Biotyper, Bruker Daltonics). Thirty-four isolates were selected for whole genome sequencing (WGS) in the Illumina MiSeq V2 platform. Sequence types (ST) were assigned by the PubMLST database (Pasteur scheme).

All but one isolate, defined as *Acinetobacter pittii*, were identified as *A. baumannii*. Susceptibility profile and PCR results are detailed in Suppl. Table. Meropenem MICs ranged from 8 to >256 mg/L (MIC_50_ = 128 mg/L, MIC_90_ > 256 mg/L). Overall, 81.7% of CRAb were resistant to at least three antimicrobial classes other than carbapenems. Resistance rateto polymyxin B was 19.1% (MIC_50_ = 1 mg/L and MIC_90_ = 4 mg/L).

**Fig 1:**
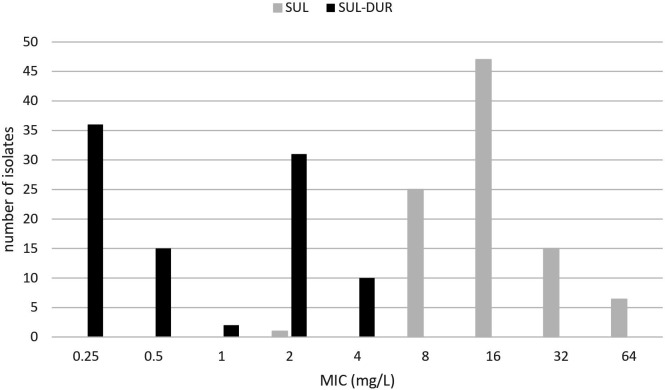
Sulbactam-durlobactam (black bars) and sulbactam (gray bars) MIC distributions for carbapenem-resistant *Acinetobacter* baumannii-calcoaceticus complex recovered from patients in Brazil.

Sulbactam MICs ranged from 2 to 64 mg/L (MIC_50_ = 16 mg/L; MIC_90_ = 32 mg/L). All *A. baumannii* (*n* = 93) were non-susceptible to sulbactam (MIC ≥ 8 mg/L), while the *A. pittii* was susceptible. Sulbactam MICs significantly reduced when adding durlobactam (Wilcoxon test: *V* = 4,371, *P* < 2.2 × 10⁻¹⁶), and susceptibility was 100% restored (MIC range 0.25–4 mg/L; MIC_50_ = 0.5 mg/L, and MIC_90_ = 4 mg/L) (Fig. 1). Of note, 10.6% of isolates exhibited borderline sulbactam-durlobactam MICs (4 mg/L).

For 96.8% of isolates, a reduction of at least two twofold dilution on MIC values was observed (Table 1). A significant increasing trend was found between sulbactam MIC and the magnitude of MIC reduction (JT = 2,193.5, *P* = 3.77 × 10⁻⁸). Baseline sulbactam MICs were positively correlated with the extent of MIC reduction (Spearman’s *ρ* = 0.55, *P* = 1.1 × 10⁻⁸).

**TABLE 1:** Association between baseline sulbactam MIC values and the magnitude of MIC reduction after the addition of durlobactam (4 mg/L)^*a*^

SUL MIC, mg/L (*n*)	Reduction of SUL MIC when adding DUR^*c*^							
	No	Onefold	Twofold	Threefold	Fourfold	Fivefold	Sixfold	Sevenfold
2 (1)	1^*b*^ (100%)	–^*d*^	–	–	–	–	–	–
*8* (25)	–	*2* (8.0%)	*10* (40.0%)	*1* (4.0%)	–	*12* (48.0%)	–	–
**16** (**47**)	–	–	**7** (14.9%)	**15** (31.9%)	**1** (2.1%)	–	**24** (51.1%)	–
**32** (**15**)	–	–	–	**1** (6.7%)	**4** (26.7%)	–	**10** (66.7%)	–
**64** (**6**)	–	–	–	–	–	**1** (16.7%)	–	**5** (83.3%)

^*a*^ SUL MIC, minimum inhibitory concentration defined by broth microdiluiton; DUR, durlobactam.

^*b*^ Isolate identified as *A. pittii* by MALDI-TOF MS.

^*c*^ Italicized values indicate isolates classified as intermediate to sulbactam alone, whereas bold gray zone indicates those resistant.

^*d*^ –, no isolates showed this reduction.

No significant differences were observed between isolates harboring *bla*_OXA-23-like_ (64.9% of the isolates) or *bla*_OXA-24/40-like_ (25.5%) regarding distribution of sulbactam MICs, sulbactam-durlobactam MICs, or the magnitude of MIC reduction after adding durlobactam (Mann–Whitney *U* tests; *P* > 0.05).

**Fig 2:**
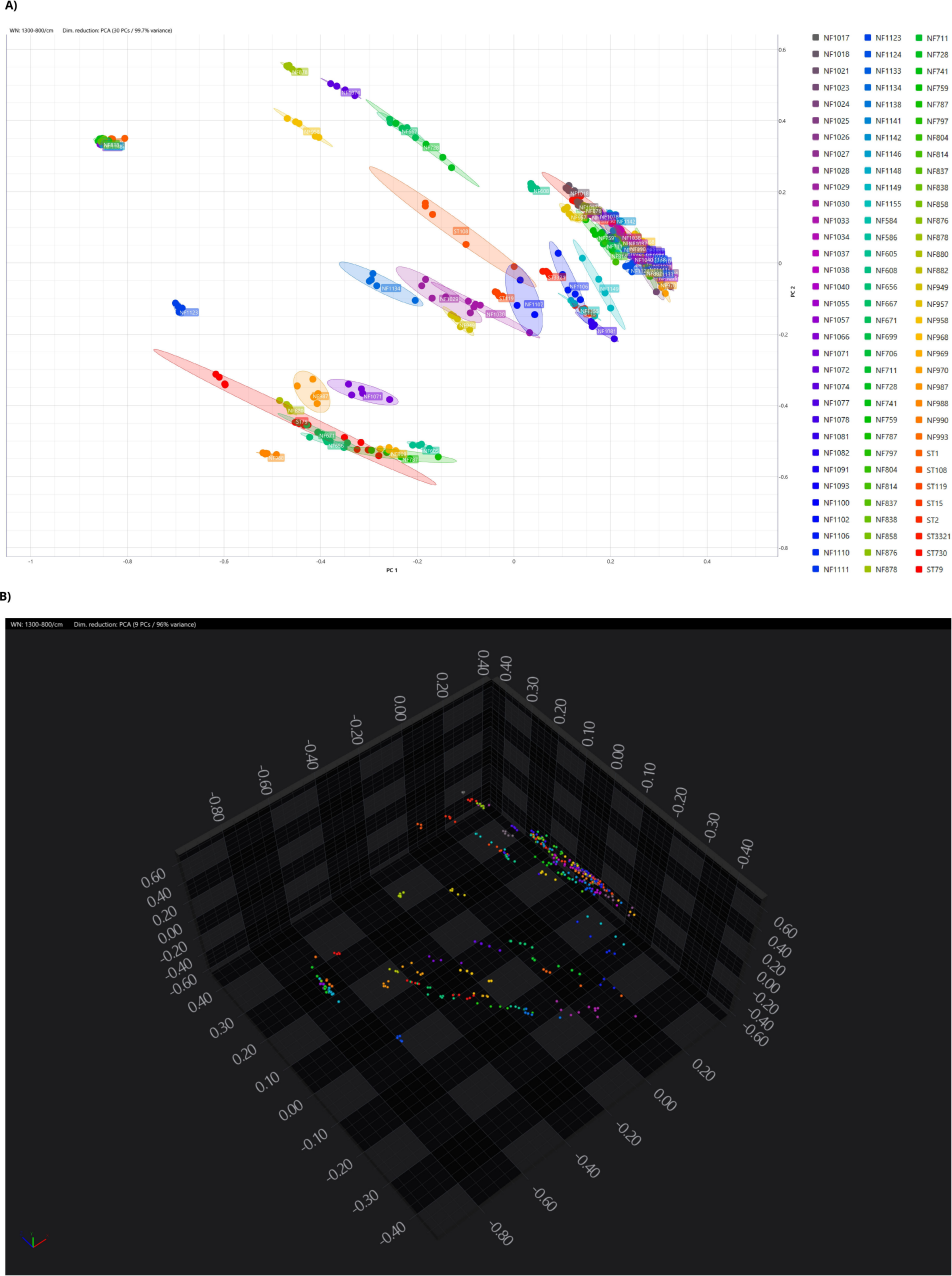
Graphical representation of IR Biotyper results (1,300–800 cm⁻¹). Five spectra were included for each isolate with a defined sequence type (ST), and at least three spectra were included for isolates without a defined ST; each color represents a single isolate. (**A**) 2D scatter plot obtained by principal component analysis (PCA) using 30 principal components, explaining 99.7% of the total variance. Ellipses indicate within-isolate dispersion, reflecting spectral similarity among replicates and discrimination between isolates along the PC1 and PC2 axes. (**B**) 3D scatter plot derived from PCA using nine principal components, accounting for 96% of the total variance.

IR Biotyper, using principal component analysis based on 30 principal components explaining 99.7% of the total variance, showed multiple distinct clusters, confirming phenotypic heterogeneity within the overall population (Fig. 2).

Table 2 presents the characteristics of isolates sequenced (33 *A. baumannii* and the *A. pittii*), including distribution of carbapenem-hydrolyzing class D β-lactamases (CHDLs) and PBP3 mutations. ST730 (41.2%) and ST79 (20.6%) were the most prevalent among the seven ST observed. A novel ST (ST3321) was identified as a single-locus variant of ST2. Almost 62% of isolates harbored *bla*_TEM-1_, which has been described as an adjuvant β-lactam resistance determinant, especially against sulbactam (22).

**TABLE 2:** Sulbactam and sulbactam-durlobactam MICs according to sequence type, OXA-carbapenemase, and cephalosporinase

Isolate	ST^*a*^	IC^*b*^	MIC, mg/L^*c*^		*bla*_OXA-23-like_	*bla*_OXA-24-like_	*bla*_OXA-51-like_	*bla*_ADC_	PBP3
			SUL	SUL-DUR					
NF584	730	5	64	0.5	–^*g*^	*bla*_OXA-72_	*bla*_OXA-65_	*bla*_ADC-183_	WT^*d*^
NF804	730	5	32	2	–	*bla*_OXA-72_	*bla*_OXA-65_	*bla*_ADC-183_	WT
NF759	730	5	16	0.25	–	*bla*_OXA-72_	*bla*_OXA-65_	*bla*_ADC-183_	WT
NF797	730	5	16	0.25	–	*bla*_OXA-72_	*bla*_OXA-65_	*bla*_ADC-183_	WT
NF608	730	5	16	1	–	*bla*_OXA-72_	*bla*_OXA-65_	*bla*_ADC-183_	WT
NF699	730	5	16	2	–	*bla*_OXA-72_	*bla*_OXA-65_	*bla*_ADC-183_	WT
NF711	730	5	16	2	–	*bla*_OXA-72_	*bla*_OXA-65_	*bla*_ADC-183_	WT
NF877	730	5	16	2	–	*bla*_OXA-72_	*bla*_OXA-65_	*bla*_ADC-183_	WT
NF949	730	5	16	2	–	*bla*_OXA-72_	*bla*_OXA-65_	*bla*_ADC-183_	WT
NF586	730	5	8	2	–	*bla*_OXA-72_	*bla*_OXA-65_	*bla*_ADC-183_	WT
NF706	730	5	8	2	–	*bla*_OXA-72_	*bla*_OXA-65_	*bla*_ADC-183_	WT
NF820	730	5	8	4	–	*bla*_OXA-72_	*bla*_OXA-65_	*bla*_ADC-183_	WT
NF983	730	5	8	4	–	*bla*_OXA-72_	*bla*_OXA-65_	*bla*_ADC-183_	WT
NF694	730	5	8	2	–	*bla*_OXA-72_	*bla*_OXA-65_	*bla*_ADC-183_	WT
NF735	79	5	64	0.5	*bla*_OXA-23_	–	*bla*_OXA-65_	*bla*_ADC-182_	WT
NF575	79	5	16	4	*bla*_OXA-23_	–	*bla*_OXA-65_	*bla*_ADC-182_	WT
NF653	79	5	16	4	*bla*_OXA-23_	–	*bla*_OXA-65_	*bla*_ADC-182_	WT
NF787	79	5	8	0.25	*bla*_OXA-23_	–	*bla*_OXA-65_	*bla*_ADC-182_	WT
NF605	79	5	8	1	*bla*_OXA-23_	–	*bla*_OXA-65_	*bla*_ADC-182_	WT
NF656	79	5	8	2	*bla*_OXA-23_	–	*bla*_OXA-65_	*bla*_ADC-182_	WT
NF671	79	5	8	2	*bla*_OXA-23_	–	*bla*_OXA-65_	*bla*_ADC-182_	WT
NF791	2	2	64	2	*bla*_OXA-23_	–	*bla*_OXA-82_	*bla*_ADC-33_	WT
NF837	2	2	16	2	*bla*_OXA-23_	–	*bla*_OXA-82_	*bla*_ADC-33_	WT
NF1112	2	2	16	2	*bla*_OXA-23_	–	*bla*_OXA-66_	*bla*_ADC-73_	A515V
NF1153	2	2	8	2	*bla*_OXA-23_	–	*bla*_OXA-66_	*bla*_ADC-33_	A515V
NF593	3321	2	16	4	*bla*_OXA-23_	–	*bla*_OXA-66_	*bla*_ADC-33_	WT
NF741	1	1	32	2	*bla*_OXA-23_	–	*bla*_OXA-69_	*bla*_ADC-181_	WT
NF838	1	1	16	2	*bla*_OXA-23_	*bla*_OXA-72_	*bla*_OXA-69_	*bla*_ADC-181_	WT
NF951	1	1	16	2	*bla*_OXA-23_	–	*bla*_OXA-69_	*bla*_ADC-181_	WT
NF667	1	1	8	0.25	*bla*_OXA-23_	–	*bla*_OXA-69_	*bla*_ADC-181_	WT
NF1145	15	3	16	2	*bla*_OXA-23_	–	*bla*_OXA-51_	*bla*_ADC-181_	A583V
NF812	15	3	8	2	*bla*_OXA-23_	–	*bla*_OXA-51_	*bla*_ADC-11_	A583V
NF809	108	1	16	0.25	*bla*_OXA-23_	–	*bla*_OXA-132_	*bla*_ADC-154_	G350S; Q488K
NF1136^*e*^	119	–	2	2	–	–	*bla*_OXA-421_ (*bla*_OXA-470-like_)^*f*^	*bla*_ADC-22_	E18G; V230I

^*a*^ ST, sequence type.

^*b*^ IC, international clone.

^*c*^ Minimum inhibitory concentration of sulbactam (SUL) and sulbactam-durlobactam (SUL-DUR) defined by broth microdilution.

^*d*^ WT, wild-type.

^*e*^ Isolate identified as *A. pittii* by MALDI-TOF MS.

^*f*^ *bla*_OXA-421 _(*bla*_OXA-470-like_).

^*g*^ –, no detected in the isolate.

Resistance to sulbactam-durlobactam has been reported (7, 12, 13) and is associated with the production of metallo-beta-lactamases (MBL) and/or mutations in PBP3 (23, 24). No MBL producers were observed.

Although most (85.3%) of our sequenced isolates exhibited wild-type PBP3, mutations in this protein (A515V, A583V, Q488K, and G350S) were also identified. Previous studies have reported the A515V mutation in both resistant and susceptible isolates (25, 26). The Q488K substitution was associated with resistance when co-occurring with Y258H (27). In our study, Q488K co-occurred with G350S.

The A583V mutation has previously been described in a ST15 *A. baumannii* from Brazil resistant to sulbactam-durlobactam that harbored *bla*_NDM-1_, introducing uncertainty regarding the specific contribution of this mutation to the phenotype (28). Our two CRAb presenting A583V mutation also belonged to ST15. Whether these mutations may facilitate the development of resistance remains to be determined.

Noteworthy, 10 isolates exhibited sulbactam-durlobactam borderline MIC, which may represent an early signal of reduced susceptibility. Five of them were sequenced, all showing a wild-type PBP3. Ongoing surveillance would be essential to understand the clinical relevance of such borderline MICs.

To the best of our knowledge, this is the second Brazilian study evaluating the *in vitro* activity of sulbactam-durlobactam. Nodari and coworkers (29) assessed the susceptibility to this antibiotic among a similar population of CRAb. They demonstrated that neither the genetic background nor the type of CHDL affected the activity of sulbactam-durlobactam, which we precisely also observed.

The most prevalent carbapenemase gene in our study was *bla*_OXA-23-like_, in line with global and regional data (30, 31). Among sequenced isolates, the OXA-24 group was represented exclusively by *bla*_OXA-72_. Indeed, it has been observed an increased frequency of OXA-72-producing *A. baumannii* in Brazil (32–34).

The integration of susceptibility profile with molecular and genomic analyses strengthens our findings. Nonetheless, this study has limitations. It was not multicentric and included a limited number of isolates, which may restrict broader extrapolation. Moreover, we did not sequence all isolates; however, phenotypic analysis using IR Biotyper confirmed marked molecular heterogeneity among CRAb isolates.

We demonstrated a consistent *in vitro* activity of sulbactam-durlobactam against a highly resistant and genetically diverse population of CRAb from Brazil. The uniform restoration of sulbactam susceptibility, regardless of carbapenemase type or genetic background, highlights the robustness of this combination supporting its role as a key therapeutic option in high-endemic settings. Given that data of this nature are virtually absent in Brazil, this study is of particular importance.

## SUPPLEMENTAL MATERIAL

Table S1 aac.00109-26-s0001.docx Characteristics of *Acinetobacter baumannii* complex isolates included in the study.

ASM does not own the copyrights to Supplemental Material that may be linked to, or accessed through, an article. The authors have granted ASM a non-exclusive, world-wide license to publish the Supplemental Material files. Please contact the corresponding author directly for reuse.
