# Supplementary material for: Consistent in vitro activity of sulbactam-durlobactam against genetically diverse carbapenem-resistant Acinetobacter baumannii in a high-endemic Brazilian setting
Source: Antimicrob Agents Chemother. 2026 Jun 12;70(7):e00109-26. doi: 10.1128/aac.00109-26 (PMC13321830; doi:10.1128/aac.00109-26)
Supplement: Table S1 — Characteristics of Acinetobacter baumannii complex isolates included in the study. [file aac.00109-26-s0001.docx]

**Suppl. Table:** Characteristics of *Acinetobacter* *baumannii* complex isolates included in the study.

| Isolate | Hospital | Date | Specimen | MIC^a^, mg/L | | | | Susceptibility profile^b^ | | | *bla*_OXA_ family^c^ |
| --- | --- | --- | --- | --- | --- | --- | --- | --- | --- | --- | --- |
|  |  |  |  | SUL | SUL-DUR | MER | POLB | CIP | SXT | AK |  |
| NF575 | H1 | Jan, 2023 | Blood | 16 | 4 | 128 | 1 | R | R | R | *bla*_OXA-23-like_ |
| NF584 | H2 | Jan, 2023 | Blood | 64 | 0.5 | >256 | 1 | R | R | R | *bla*_OXA-24-like_ |
| NF586 | H1 | Jan, 2023 | Blood | 8 | 2 | 256 | 1 | R | R | R | *bla*_OXA-24-like_ |
| NF593 | H3 | Mar, 2023 | Blood | 16 | 4 | 128 | 16 | R | R | R | *bla*_OXA-23-like_ |
| NF605 | H2 | Mar, 2023 | Blood | 8 | 1 | 64 | 0.5 | R | R | R | *bla*_OXA-23-like_ |
| NF608 | H1 | Mar, 2023 | Blood | 16 | 1 | >256 | 0.5 | R | R | R | *bla*_OXA-24-like_ |
| NF653 | H1 | Aug, 2023 | Blood | 16 | 4 | 64 | 1 | R | R | R | *bla*_OXA-23-like_ |
| NF656 | H1 | Aug, 2023 | Blood | 8 | 2 | 64 | 0.5 | R | R | R | *bla*_OXA-23-like_ |
| NF667 | H3 | Sep, 2023 | Blood | 8 | 0.25 | 64 | 32 | R | I | R | *bla*_OXA-23-like_ |
| NF671 | H1 | Sep, 2023 | Blood | 8 | 2 | 128 | 1 | R | R | R | *bla*_OXA-23-like_ |
| NF694 | H2 | Sep, 2023 | Blood | 8 | 2 | >256 | 0.5 | R | R | R | *bla*_OXA-24-like_ |
| NF699 | H1 | Sep, 2023 | Blood | 16 | 2 | 256 | 0.5 | R | R | R | *bla*_OXA-24-like_ |
| NF706 | H2 | Sep, 2023 | Blood | 8 | 2 | >256 | 0.5 | R | R | R | *bla*_OXA-24-like_ |
| NF711 | H1 | Sep, 2023 | Blood | 16 | 2 | >256 | 0.5 | R | R | R | *bla*_OXA-24-like_ |
| NF728 | H1 | Fev, 2024 | Blood | 8 | 2 | 64 | 1 | R | R | R | *bla*_OXA-23-like_ |
| NF735 | H1 | Fev, 2024 | Blood | 64 | 0.5 | 64 | 1 | R | R | R | *bla*_OXA-23-like_ |
| NF741 | H1 | Fev, 2024 | Blood | 32 | 2 | 128 | 4 | R | R | R | *bla*_OXA-23-like_ |
| NF759 | H1 | Fev, 2024 | Blood | 16 | 0.25 | 256 | 1 | R | R | R | *bla*_OXA-24-like_ |
| NF787 | H1 | Mar, 2024 | Blood | 8 | 0.25 | 128 | 1 | R | R | R | *bla*_OXA-23-like_ |
| NF791 | H1 | Mar, 2024 | Blood | 64 | 2 | 128 | 1 | R | R | R | *bla*_OXA-23-like_ |
| NF797 | H1 | Mar, 2024 | Blood | 16 | 0.25 | >256 | 1 | R | R | R | *bla*_OXA-24-like_ |
| NF804 | H1 | Mar, 2024 | Blood | 32 | 2 | >256 | 0.5 | R | R | R | *bla*_OXA-24-like_ |
| NF809 | H1 | Mar, 2024 | Blood | 16 | 0.25 | 64 | 16 | I | S | R | *bla*_OXA-23-like_ |
| NF812 | H1 | Mar, 2024 | Blood | 8 | 2 | 64 | 2 | R | R | R | *bla*_OXA-23-like_ |
| NF814 | H1 | Mar, 2024 | Blood | 32 | 0.5 | >256 | 2 | R | R | R | *bla*_OXA-23-like_ |
| NF820 | H1 | Mar, 2024 | Blood | 8 | 4 | 128 | 1 | R | R | R | *bla*_OXA-24-like_ |
| NF837 | H1 | Apr, 2024 | Blood | 16 | 2 | 128 | 1 | R | R | R | *bla*_OXA-23-like_ |
| NF838 | H1 | Apr, 2024 | Blood | 16 | 2 | 128 | 1 | R | R | R | *bla*_OXA-23-like_; *bla*_OXA-24-like_ |
| NF858 | H2 | Apr, 2024 | Other^e^ | 32 | 2 | 256 | 1 | R | R | R | *bla*_OXA-24-like_ |
| NF876 | H1 | Dec, 2024 | Blood | 16 | 2 | >256 | 0.5 | R | R | R | *bla*_OXA-23-like_ |
| NF877 | H1 | Dec, 2024 | Blood | 16 | 2 | >256 | 0.5 | R | R | R | *bla*_OXA-24-like_ |
| NF878 | H2 | Dec, 2024 | Traqueal aspirate | 16 | 4 | 64 | 32 | R | R | R | *bla*_OXA-23-like_ |
| NF880 | H1 | Dec, 2024 | Traqueal aspirate | 16 | 2 | 128 | 4 | R | R | R | *bla*_OXA-23-like_ |
| NF882 | H2 | Dec, 2024 | Traqueal aspirate | 32 | 0.5 | 256 | 0.5 | R | S | S | *bla*_OXA-23-like_ |
| NF949 | H1 | Dec, 2024 | Blood | 16 | 2 | 128 | 1 | R | R | R | *bla*_OXA-24-like_ |
| NF951 | H1 | Dec, 2024 | Blood | 16 | 2 | 128 | 32 | R | R | R | *bla*_OXA-23-like_ |
| NF957 | H1 | Dec, 2024 | Sputum | 16 | 4 | 256 | 2 | R | R | R | *bla*_OXA-24-like_ |
| NF958 | H1 | Dec, 2024 | Traqueal aspirate | 16 | 2 | 64 | 2 | R | R | R | *bla*_OXA-23-like_ |
| NF968 | H2 | Dec, 2024 | Traqueal aspirate | 16 | 2 | 128 | 2 | R | R | R | *bla*_OXA-23-like_ |
| NF969 | H2 | Dec, 2024 | Traqueal aspirate | 16 | 0.25 | 64 | 2 | R | R | R | *bla*_OXA-23-like_ |
| NF970 | H2 | Dec, 2024 | Traqueal aspirate | 32 | 2 | 128 | 0.5 | R | R | S | *bla*_OXA-23-like_ |
| NF983 | H2 | Dec, 2024 | Blood | 8 | 4 | >256 | 1 | R | R | S | *bla*_OXA-24-like_ |
| NF987 | H1 | Dec, 2024 | Traqueal aspirate | 16 | 0.25 | 64 | 1 | R | R | R | *bla*_OXA-23-like_ |
| NF988 | H1 | Dec, 2024 | Sputum | 8 | 0.25 | 64 | 1 | R | R | R | *bla*_OXA-23-like_ |
| NF990 | H1 | Dec, 2024 | Sputum | 8 | 0.25 | 64 | 1 | R | S | S | *bla*_OXA-23-like_ |
| NF993 | H2 | Dec, 2024 | Traqueal aspirate | 64 | 0.5 | 128 | 1 | R | S | S | *bla*_OXA-23-like_ |
| NF1017 | H1 | Jan, 2025 | Urine | 8 | 0.25 | 256 | 16 | R | R | R | *bla*_OXA-24-like_ |
| NF1018 | H2 | Jan, 2025 | Traqueal aspirate | 8 | 0.25 | 128 | 1 | R | R | R | *bla*_OXA-23-like_ |
| NF1021 | H2 | Jan, 2025 | Urine | 32 | 0.5 | 128 | 1 | R | I | S | *bla*_OXA-23-like_ |
| NF1023 | H1 | Jan, 2025 | Sputum | 32 | 4 | 256 | 1 | R | I | S | *bla*_OXA-23-like_ |
| NF1024 | H2 | Jan, 2025 | Traqueal aspirate | 16 | 4 | 128 | 1 | R | R | R | *bla*_OXA-23-like_ |
| NF1025 | H2 | Jan, 2025 | Cerebrospinal fluid | 16 | 4 | 128 | 4 | R | R | R | *bla*_OXA-23-like_ |
| NF1026 | H2 | Jan, 2025 | Traqueal aspirate | 32 | 0.5 | >256 | 1 | R | R | R | *bla*_OXA-24-like_ |
| NF1027 | H1 | Jan, 2025 | Traqueal aspirate | 16 | 0.25 | >256 | 1 | R | R | R | *bla*_OXA-23-like_ |
| NF1028 | H1 | Jan, 2025 | Traqueal aspirate | 16 | 2 | 256 | 1 | R | R | S | *bla*_OXA-23-like_ |
| NF1029 | H1 | Jan, 2025 | Urine | 16 | 2 | 64 | 2 | R | R | R | *bla*_OXA-23-like_ |
| NF1030 | H1 | Jan, 2025 | Traqueal aspirate | 16 | 0.25 | 64 | 4 | R | R | R | *bla*_OXA-23-like_ |
| NF1033 | H2 | Jan, 2025 | Traqueal aspirate | 16 | 0.25 | 128 | 4 | R | R | R | *bla*_OXA-23-like_ |
| NF1034 | H2 | Jan, 2025 | Traqueal aspirate | 16 | 0.25 | >256 | 1 | R | R | S | *bla*_OXA-24-like_ |
| NF1037 | H1 | Jan, 2025 | Traqueal aspirate | 8 | 0.25 | 64 | 4 | R | R | R | *bla*_OXA-23-like_ |
| NF1038 | H1 | Jan, 2025 | Blood | 16 | 0.25 | 128 | 4 | R | I | S | *bla*_OXA-23-like_ |
| NF1040 | H2 | Jan, 2025 | Traqueal aspirate | 16 | 0.25 | 128 | 1 | R | R | R | *bla*_OXA-23-like_ |
| NF1055 | H2 | Jan, 2025 | Bronchoalveolar lavage | 16 | 0.25 | >256 | 2 | R | R | R | *bla*_OXA-24-like_ |
| NF1057 | H2 | Jan, 2025 | Urine | 32 | 0.5 | 64 | 2 | R | R | R | *bla*_OXA-23-like_ |
| NF1066 | H2 | Jan, 2025 | Other | 8 | 0.25 | 128 | 2 | R | R | R | *bla*_OXA-23-like_ |
| NF1071 | H2 | Jan, 2025 | Other | 32 | 0.5 | 64 | 1 | R | R | R | *bla*_OXA-23-like_; *bla*_OXA-24-like_ |
| NF1072 | H1 | Jan, 2025 | Traqueal aspirate | 32 | 0.5 | >256 | 8 | R | R | R | *bla*_OXA-23-like_; *bla*_OXA-24-like_ |
| NF1074 | H1 | Jan, 2025 | Traqueal aspirate | 16 | 0.25 | 64 | 2 | R | R | R | *bla*_OXA-23-like_ |
| NF1077 | H1 | Jan, 2025 | Traqueal aspirate | 8 | 0.25 | 128 | 0.5 | R | R | R | *bla*_OXA-23-like_ |
| NF1078 | H2 | Jan, 2025 | Bronchoalveolar lavage | 8 | 0.25 | >256 | 0.5 | R | R | R | *bla*_OXA-24-like_ |
| NF1081 | H1 | Jan, 2025 | Traqueal aspirate | 16 | 0.25 | 64 | 1 | R | S | S | *bla*_OXA-23-like_ |
| NF1082 | H1 | Jan, 2025 | Other | 16 | 0.25 | 128 | 0.5 | R | R | R | *bla*_OXA-24-like_ |
| NF1091 | H2 | Feb, 2025 | Blood | 64 | 0.5 | 128 | 0.5 | R | R | R | *bla*_OXA-23-like_ |
| NF1093 | H2 | Feb, 2025 | Traqueal aspirate | 32 | 0.5 | 64 | 0.5 | R | R | R | *bla*_OXA-23-like_ |
| NF1100 | H2 | Feb, 2025 | Cerebrospinal fluid | 16 | 0.25 | 128 | 0.5 | R | R | R | *bla*_OXA-23-like_ |
| NF1102 | H1 | Feb, 2025 | Traqueal aspirate | 8 | 0.25 | 8 | 2 | I | S | S | None^f^ |
| NF1106 | H1 | Feb, 2025 | Blood | 8 | 2 | 8 | 0.25 | I | S | S | None |
| NF1110 | H2 | Feb, 2025 | Rectal swab | 16 | 0.25 | 64 | 2 | R | R | R | *bla*_OXA-23-like_ |
| NF1111 | H2 | Feb, 2025 | Traqueal aspirate | 8 | 0.25 | 256 | 1 | R | R | R | *bla*_OXA-24-like_ |
| NF1112 | H2 | Feb, 2025 | Blood | 16 | 2 | 128 | 0.5 | R | R | R | *bla*_OXA-23-like_ |
| NF1123 | H2 | Feb, 2025 | Urine | 32 | 0.5 | 128 | 4 | R | R | R | *bla*_OXA-23-like_ |
| NF1124 | H2 | Feb, 2025 | Urine | 16 | 0.25 | 256 | 2 | R | R | R | *bla*_OXA-24-like_ |
| NF1133 | H2 | Feb, 2025 | Bronchoalveolar lavage | 8 | 2 | 256 | 1 | R | R | R | *bla*_OXA-23-like_; *bla*_OXA-24-like_ |
| NF1134 | H1 | Feb, 2025 | Traqueal aspirate | 16 | 0.25 | 32 | 2 | R | R | R | None |
| NF1136^g^ | H1 | Feb, 2025 | Blood | 2 | 2 | 8 | 0.5 | I | S | S | None |
| NF1138 | H1 | Feb, 2025 | Sputum | 16 | 0.25 | 64 | 1 | R | R | R | *bla*_OXA-23-like_ |
| NF1141 | H1 | Feb, 2025 | Traqueal aspirate | 16 | 0.25 | 128 | 2 | R | R | R | *bla*_OXA-23-like_ |
| NF1142 | H1 | Feb, 2025 | Sputum | 16 | 0.25 | 64 | 0.5 | R | R | R | *bla*_OXA-23-like_ |
| NF1145 | H1 | Feb, 2025 | Blood | 16 | 2 | 64 | 0.25 | R | S | R | *bla*_OXA-23-like_ |
| NF1146 | H1 | Feb, 2025 | Traqueal aspirate | 32 | 0.5 | 128 | 8 | R | R | R | *bla*_OXA-23-like_ |
| NF1148 | H1 | Feb, 2025 | Other | 64 | 0.5 | 128 | 8 | R | R | R | *bla*_OXA-23-like_ |
| NF1149 | H1 | Feb, 2025 | Urine | 16 | 0.25 | 16 | 4 | I | S | S | None |
| NF1153 | H1 | Feb, 2025 | Blood | 8 | 2 | 64 | 0.5 | R | R | R | *bla*_OXA-23-like_ |
| NF1155 | H1 | Feb, 2025 | Traqueal aspirate | 16 | 0.25 | 128 | 2 | R | R | R | *bla*_OXA-23-like_ |

^a^ minimum inhibitory concentration of sulbactam (SUL), sulbactam-durlobactam (SUL-DUR), meropenem (MER) and POLB (polymyxin B) determined by broth microdilution;

^b^ Determined by disk diffusion, according to the routine of hospital’s clinical microbiology laboratories. CIP, ciprofloxacin (5μg); SXT, trimethoprim- sulfamethoxazole (1.25/23.75 µg); AK, amikacin (30 µg);

^c^ Results of multiplex PCR for *bla*_OXA-23-like_ and *bla*_OXA-24-like_;

^d^ Not defined;

^e^ Other clinical specimens rarely observed;

^f^ No amplification on multiplex reaction;

^g^Isolate identified as *Acinetobacter pittii* by MALDI-TOF MS.
